# Angiotensin A/Alamandine/MrgD Axis: Another Clue to Understanding Cardiovascular Pathophysiology

**DOI:** 10.3390/ijms17071098

**Published:** 2016-07-20

**Authors:** Jaroslav Hrenak, Ludovit Paulis, Fedor Simko

**Affiliations:** 1 Institute of Pathophysiology, Faculty of Medicine, Comenius University, 811 08 Bratislava, Slovakia; jaro.hrenak@gmail.com (J.H.); ludovit.paulis@gmail.com (L.P.); 2 1st Clinic of Medicine, Donauisar Klinikum, 944 69 Deggendorf, Germany; 3 Institute of Normal and Pathological Physiology, Slovak Academy of Sciences, 814 38 Bratislava, Slovakia; 4 3rd Clinic of Medicine, Faculty of Medicine, Comenius University, 833 05 Bratislava, Slovakia; 5 Institute of Experimental Endocrinology, BMC, Slovak Academy of Sciences, 814 38 Bratislava, Slovakia

**Keywords:** angiotensin A, alamandine, MrgD receptor, renin-angiotensin system

## Abstract

The renin-angiotensin system (RAS) plays a crucial role in cardiovascular regulations and its modulation is a challenging target for the vast majority of cardioprotective strategies. However, many biological effects of these drugs cannot be explained by the known mode of action. Our comprehension of the RAS is thus far from complete. The RAS represents an ingenious system of “checks and balances”. It incorporates vasoconstrictive, pro-proliferative, and pro-inflammatory compounds on one hand and molecules with opposing action on the other hand. The list of these molecules is still not definitive because new biological properties can be achieved by minor alteration of the molecular structure. The angiotensin A/alamandine-MrgD cascade associates the deleterious and protective branches of the RAS. Its identification provided a novel clue to the understanding of the RAS. Angiotensin A (Ang A) is positioned at the “crossroad” in this system since it either elicits direct vasoconstrictive and pro-proliferative actions or it is further metabolized to alamandine, triggering opposing effects. Alamandine, the central molecule of this cascade, can be generated both from the “deleterious” Ang A as well as from the “protective” angiotensin 1–7. This pathway modulates peripheral and central blood pressure regulation and cardiovascular remodeling. Further research will elucidate its interactions in cardiovascular pathophysiology and its possible therapeutic implications.

## 1. Introduction

The renin-angiotensin system (RAS) plays a crucial role in pathophysiological cardiovascular conditions, such as hypertension, heart failure, endothelial dysfunction, and atherosclerosis development [1,2,3,4]. Nowadays, the RAS can be pharmacologically modulated at several levels and its inhibition has been well established in the clinical practice. It is generally accepted that the effects of angiotensin-converting enzyme (ACE) inhibitors, angiotensin type 1 receptor (AT1) blockers, direct renin inhibitors, and aldosterone receptor antagonists provide protection beyond blood pressure (BP) reduction in addition to their hemodynamic effects [4,5,6,7,8,9]. The pleiotropic nature of these drugs involves inhibition of pathological proliferation, aggregation, coagulation, inflammation, and neurohumoral activation at different molecular levels, resulting in prevention or reversion of cardiac and vascular remodeling and preserving aerobic metabolism in the heart, brain, and peripheral organs. Most importantly, these interventions are associated with a reduction of cardiovascular events and mortality.

**Figure 1:**
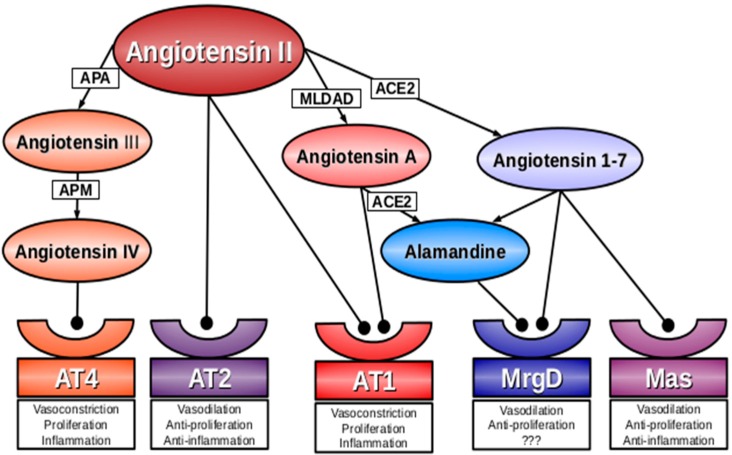
The position of the angiotensin A/alamandine/MrgD signaling pathway within the renin-angiotensin system. The “deleterious” molecules are marked in red/orange, the “protective” ones are marked in blue/purple. ACE2—angiotensin-converting enzyme type 2; AT1, AT2, AT3—angiotensin receptor type 1, 2 and 3, respectively, APA—aminopeptidase A; APM—aminopeptidase M; MLDAD—mononuclear leukocyte-derived aspartate decarboxylase.

Although our knowledge on the RAS has substantially increased in recent decades, a number of biological effects cannot be satisfactorily explained by the well-established mechanisms of action. This fact indicates that our present understanding of the RAS is far from complete. There are several blank spaces in our concept of the RAS and its role in cardiovascular regulation. First, the system represents more than a simple signaling pathway. Many new members of the RAS-modulating family have been identified and their list is probably still not definitive. A small change in the molecular structure can create novel, original properties defining the biological implications of a particular compound. Second, the generation of RAS peptides comprises several collateral, mutually interfering, enzymatic pathways, which makes the final effect of a pharmacological intervention difficult to predict. Third, the RAS incorporates molecules with opposing biological actions. One line is represented by vasoconstrictive, pro-proliferative, and pro-inflammatory molecules, such as ACE, angiotensin II (Ang II), and AT1 receptors. The other line involves ACE2, angiotensin 1–7 (Ang 1–7), angiotensin type 2 (AT2) receptor, Mas receptor or *N*-acetyl-seryl-aspartyl-lysyl-proline (Ac-SDKP) [8,9,10,11], which exert effects at least partially counteracting the potentially harmful actions of the deleterious arm of the RAS (Figure 1).

Besides the classic endocrine RAS, the components of the system have been identified in a variety of mammalian tissues. The existence of the local (tissue) RAS with autocrine or paracrine actions underlies the fact that a stimulation or inhibition of the RAS can induce variable biological effects in different tissues [4,6,11,12,13,14,15].

Considering this complexity of the RAS, the available therapeutic compounds are likely to interfere with a number of RAS components. Such cross-reaction makes the prediction of the therapeutic outcome much more difficult. Increasing incidence and prevalence of cardiovascular diseases and their target organ complications creates a continuous need for novel therapeutic options [1,7,14,15,16]. Their development requires a deeper understanding of the mechanisms leading to hypertension and related structural and functional alteration of the heart, blood vessels, and kidneys. In order to improve the therapeutic benefit of antihypertensive agents, it is desirable to disclose the key players and their biological impact.

The recently identified signaling cascade comprising angiotensin A/alamandine/MrgD represents a new, previously missing, piece in the RAS puzzle. Although only little is known about this pathway and its biological and pathophysiological relevance is unclear, it may represent another step to understanding the complexity of the RAS. This review is focused on this novel signaling cascade with the aim to deepen the insight of researchers and clinicians into this tricky system.

## 2. Angiotensin A

**Figure 2:**
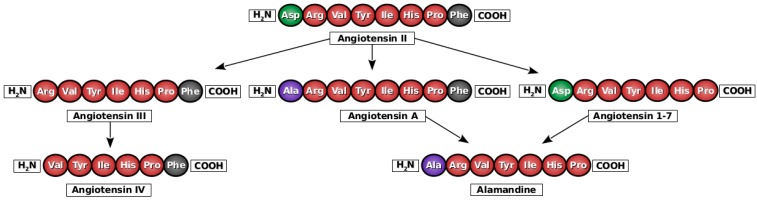
Amino acid sequences of angiotensin II, angiotensin III, angiotensin IV, angiotensin A, angiotensin 1–7 and alamandine. A single change in one amino acid can create novel properties.

In 2007, Jankowski et al. identified a novel vasoconstrictive angiotensin-derived octapeptide in human plasma and he named it angiotensin A (Ang A), where A stands for alanine. The amino acid sequence of this peptide (Ala–Arg–Val–Tyr–Ile–His–Pro–Phe) differs from Angiotensin II (Ang II) only in one amino acid: Ala^1^ instead of Asp^1^ (Figure 2) [17].

This molecule represents a biologically active metabolite of Ang II, which can be further converted to another active peptide alamandine. It is physiologically present in human plasma at concentrations lower than 20% of normal Ang II concentrations. However, the Ang A/II ratio is significantly increased in end-stage renal disease patients. Ang A is supposedly generated from Ang II by decarboxylation of Asp^1^. This pathway was originally demonstrated in human mononuclear lymphocytes, which indicates the involvement of mononuclear leukocyte-derived aspartate decarboxylase [17].

Ang A interacts with both main types of angiotensin receptors AT1 and AT2. While Ang A and Ang II do not differ in their affinity to AT1 receptor, the affinity of Ang A to the AT2 receptor was shown to be significantly higher than that of Ang II [17]. Yet, this finding has been disputed by another group suggesting similar AT2 receptor affinity for Ang II and Ang A [18].

The available data suggest that similarly to Ang II the Ang A induces vasoconstriction, although with substantially lower potency compared to Ang II. In order for Ang A to elicit the same maximal vasoactive effect as Ang II 10-fold higher concentrations are required compared to Ang II [17,18]. Administration of Ang A was not associated with any vasodilatory effect, not even after AT1 receptor blockade [18]. In contrast to Ang II, the Ang A-mediated vasoconstriction was significantly lower in the abdominal aorta from rabbits fed with atherogenic diet, leading to speculations that this decrease in vasoactive response might be compensated by increased plasma concentrations of Ang A in certain pathological conditions [19].

Experiments on isolated perfused rat kidneys revealed that the vasoconstrictive effect of Ang A could be inhibited by AT1 receptor antagonist EXP-3174 (active metabolite of losartan), whereas AT2 receptor antagonist PD123319 had no effect [17]. These findings were confirmed in vivo in normotensive and spontaneously hypertensive rats using ultrasound assessment of renal blood flow [18]. Intravenous or intrarenal application of Ang A resulted in decreased renal perfusion and increased the mean arterial pressure. Inhibition of AT1 signaling with candesartan reversed the effect of Ang A, whereas the AT2 receptor antagonist PD123319 did not affect the renal perfusion [18].

The vasoconstrictive effect of Ang A translates into the enhancement of arterial BP. Intravenous administration of Ang A resulted in a significant dose-dependent increase in BP in several experiments [18,19,20,21]. However, significantly higher concentrations of Ang A compared to Ang II were needed to achieve the same level of BP increase [17,18]. Analogically to the previous studies on isolated perfused kidneys, also the Ang A-induced increase in systolic BP was antagonized by AT1 receptor-blockers, candesartan or losartan, in vivo [18,20,21] and PD123319 failed to attenuate the vasopressor activity of Ang A [18,20].

Experiments on AT1 receptor knockout and wild type mice showed that administration of Ang A to mice expressing no AT1 receptors was not associated with any significant increase in BP [17]. These data are in accordance with the previous findings that the effect of Ang A can be abolished by AT1 receptor blockers and indicate that the vasopressor effect of both the Ang A and the Ang II is mediated via the AT1 receptor.

In isolated perfused hearts, Ang A substantially reduced coronary flow [21]. Whereas the administration of Ang II led to prolonged duration of ventricular tachyarrhythmias and/or ventricular fibrillation in the reperfusion phase, the effect of Ang A was not statistically significant. Interestingly, contrary to Ang II, Ang A did not affect the amplitude of the calcium transient in isolated ventricular cardiomyocytes [21]. Moreover, in comparison to Ang II, Ang A exerted a greater in vitro pro-proliferative effect on rat vascular smooth muscle cells [20]. While the vasoconstrictive potency of Ang A was absent in AT1-knock-out animals and completely abolished by AT1 receptor blockers, the effect of Ang A on the heart seems to be only partly mediated via AT1 receptors. Therefore, a distinct intracellular signaling cascade for Ang A in cardiomyocytes is to be anticipated [21].

To summarize, Ang A exerts vasoconstrictive and pro-hypertensive effects, which seem to be AT1 receptor mediated (Table 1). Although Ang A can also bind to AT2 receptors and its affinity to this receptor is at least the same or even higher compared to Ang II, the role of this interaction in physiological and pathological conditions remains unclear. An interesting question is whether Ang A could induce physiological effects of AT2 receptor stimulation in the presence of AT1 receptor blockade such as natriuresis, neuroprotection, or inhibition of fibrosis. Further research may disclose the potential effect of Ang A on aldosterone metabolism or fibrosis development as well as to disclose the molecular mechanisms underlying these actions.

## 3. Alamandine

Recently, a new component of the RAS called alamandine was identified by mass spectrometry [22]. By now, the peptide was detected in rats, mice as well as in humans. Similarly to the previous findings on Ang A, alamandine plasma levels were increased in patients with renal disease. Alamandine is a heptapeptide with amino acid sequence Ala–Arg–Val–Tyr–Ile–His–Pro, differing from Ang (1–7) only in the N-terminal alanine instead of aspartate residue (Figure 2). It was postulated that alamandine is generated by catalytic hydrolysis of Ang A by the action of angiotensin-converting enzyme 2 (ACE2) or directly from Angiotensin 1–7 (Ang 1–7) by decarboxylation of its aspartate residue [22].

In aortic rings from FVB/N mice, alamandine induced endothelial-dependent vasorelaxation similar to the effect of Ang 1–7 [22,23]. However, contrary to Ang 1–7, alamandine can counteract even the vasoconstriction induced by its precursor Ang A without affecting Ang II-induced vasoconstriction [19]. The investigation of vasoreactivity on rabbit vessels indicated that the vasodilatory effect of alamandine was mediated by acetylcholine. The presence of alamandine increased the bioavailability of acetylcholine and it promoted acetylcholine-mediated vasodilation in the thoracic aorta and iliac artery. However, in the carotid artery, alamandine affected neither acetylcholine levels nor the vascular tone. In the renal artery, it even suppressed the acetylcholine-induced vasodilation. Yet, in the arteries obtained from animals fed with atherogenic diet alamandine elicited no significant vasoactive effects [19].

Surprisingly, alamandine induced vasorelaxation in Mas-knockout mice [22], indicating a signaling pathway distinct from the Ang 1–7/Mas axis. In agreement with this finding, the effect of alamandine was not abolished by Mas receptor antagonist A-779 but it was prevented by the Ang 1–7 antagonist d-Pro^7^-Ang 1–7. Alamandine induced NO-release in MrgD-transfected cells but not in Mas-transfected cells, suggesting that alamandine was a natural ligand for MrgD receptor [22,24]. In AT2 wild type animals, the vasoactive effect of alamandine was antagonized by AT2 receptor antagonist PD123319 but it was present in AT2-knockout mice. Surprisingly, PD123319 blocked alamandine effects in AT2-knockouts as well. These findings suggest a different mechanism of action of alamandine than the interaction with AT2 receptor and they indicate that PD123319 in vivo antagonized the binding of alamandine to MrgD-transfected cells. These data confirm the low selectivity of PD123319, which beside the AT2 (and in higher concentrations AT1) receptor blocks the MrgD receptor as well [22].

Spontaneously hypertensive rats, after receiving a single dose of alamandine/β-hydroxypropyl cyclodextrin compound, showed a long-term BP reduction [22]. The effect of alamandine on BP is probably more complex and next to the peripheral vascular tone regulations it also involves central regulatory mechanisms. A microinjection of alamandine into the rostral ventrolateral medulla of rats induced a vasopressor effect, whereas an administration into the caudal ventrolateral medulla elicited a vasodepressor effect [22]. In addition, an intracerebral ventricular infusion of alamandine potentiated the bradycardic component of the baroreflex [24].

Alamandine seems also to exert antiremodeling effects. A chronic administration of alamandine to isoproterenol-treated Wistar rats was associated with a decreased accumulation of collagen I, collagen III, and fibronectin in the heart [22,25].

Biological effects of alamandine are listed in Table 2.

## 4. Mas-Related G-Protein Coupled Receptor D (MrgD)

In 2001–2002, two research teams in parallel described novel receptors belonging to the G protein-coupled receptor family. Those receptors were related to *mas* protooncogene and they were named Mrg [26] or sensory neuron-specific G-protein coupled receptors (SNSR) [27]. Based on sequence homology, Mrgs were further divided into several subfamilies, e.g., MrgA-H, MrgX1-7, Mas1, *etc.*, identified both in experimental animals and humans [28]. At the same time, Ang 1–7 was identified as a natural endogenous ligand for the Mas receptor [29]. Moreover, Ang 1–7, Ang III and IV were able to induce the release of arachidonic acid in response to the stimulation of several receptors of the Mrg family including MrgD [30]. These findings posed a question on the possible interaction between the Mrg receptor family and the RAS.

Mas-related G-protein coupled receptors have been originally identified in primary nociceptive sensory neurons in rodents and humans [26,27]. Accordingly, MrgD receptors were found in the dorsal root ganglia [31] participating in enhanced neuronal excitability [32]. It is suggested that they play a role in the modulation of neuropathic pain. However, MrgD receptors were identified in other tissues, such as testis, urinary bladder, arteries, uterus, skin, cerebellum, trachea, thymus, heart, lung, diaphragm, skeletal muscle, prostate, seminal vesicle, and white and brown adipose tissue [33,34]. The expression of MgrD was reported in association with a number of pathologies, e.g., inflammatory bowel disease [34], atherosclerotic aorta [19], or lung cancer [35]. Using immunohistochemical analysis, MrgDs were identified within atherosclerotic plaques, in smooth muscle cells and in endothelial cells expressing endothelial nitric oxide (NO) synthase (eNOS) [19].

Mas-related G-protein coupled receptor D was initially reported as a receptor for β-alanine [31]. Uno et al. [36] revealed two more physiological ligands for MrgD: the β-aminoisobutyric acid (βAIBA) and diethylstilbestrol (DES). Most recently, it was shown that MrgD can be activated by Ang 1–7 signaling, and this signaling cascade involves adenylyl cyclase, cAMP, and proteinkinase A [30,37]. A high degree of amino acid sequence homology between Ang 1–7 and alamandine triggered speculations that alamandine might interact with the Mas receptor (the primary known receptor for Ang 1–7) and/or with Mrgs. Indeed, in vitro experiments with MrgD- and Mas-transfected cells indicated that MrgD might be a natural endogenous receptor for alamandine. MrgD-transfected cells, contrary to Mas-transfected cells, reacted to stimulation by alamandine with NO-release [22]. In agreement with the above findings, alamandine elicited endothelium-dependent vasorelaxation of aortic rings, whereas the presence of β-alanine (another ligand for MrgD) in the incubation medium did not induce any vasoactive response and it even inhibited the alamandine-induced vasorelaxation [20]. On the other hand, the stimulation with β-alanine resulted in other biological effects in terms of nociception and itch [38,39].

It is obvious that MrgD receptors are the target for variable ligands in different tissues resulting in distinct biological effects. The recently discovered interactions of alamandine and Ang 1–7 with MrgD receptor suggest that the role of MrgD-mediated signaling in the RAS is more complex than presumed and considerations regarding the potential role of this pathway in cardiovascular pathophysiology are justifiably emerging.

## 5. Conclusions

The identification of the Ang A/alamandine-MrgD signaling cascade is the most recent step in understanding the complexity of the RAS and its role in cardiovascular physiology and pathology. This signaling pathway associates with both the “deleterious” as well with the “protective” RAS axis. Ang A is positioned at a “crossroad” in the system, since it can either directly elicit vasoconstrictive and pro-proliferative actions or indirectly trigger opposing effects after being further metabolized to alamandine. Alamandine can be regarded as the central molecule of this signaling cascade. Alamandine seems to antagonize Ang A-induced effects leading to a negative feedback loop. Alamandine can be generated both from the “deleterious” Ang A as well as from the “protective” Ang 1–7.

The here-described novel molecular pathway might participate in peripheral and central BP regulation and cardiovascular remodeling. We have to admit that our knowledge on this cascade is just emerging, and that only further research can elucidate its role in cardiovascular physiology, pathology, or even therapeutic targeting.

## Figures and Tables

**Table 1:** Bilogical effects of Angiotensin A.

Angiotensin A		References
Human embryonic kidney cells HEK-293	No difference in AT1 affinity to Ang A and Ang II ↑ affinity of AT2 for Ang A than for Ang II	[17]
Vascular smooth-muscle cells	Dose-dependent ↑ in cytosolic calcium inhibited by AT1 antagonist EXP-3174	[17]
	proliferative effect Ang A > to Ang II	[20]
Abdominal aorta New Zealand White rabbits	Vasoconstriction ↓ in vessels from animals fed with atherogenic diet	[19]
Isolated perfused kidney	Dose-dependent vasoconstriction 90% of the maximal effect of Ang II inhibited by AT1 antagonist EXP-3174 no effect of AT2 antagonist PD123319	[17]
Normotensive rats intrarenal administration	↓ renal blood flow and ↑ renal vascular resistance ↓ effect of Ang A compared to Ang II improved by candesartan	[18]
Normotensive rats i.v. administration	↑ BP ↓ by AT1-receptor blocker losartan no effect of AT2-antagonist PD123319	[20]
Spontaneously hypertensive rats i.v. administration	↑ BP both SHR and controls ↓ by AT1-receptor blocker candesartan no effect of AT2-antagonist PD123319 dose-dependent ↓ renal blood flow and ↑ renal vascular resistance in both SHR and controls ↓ effect of Ang A compared to Ang II no vasodilator response to Ang A or Ang II stimulation improved by candesartan no effect of AT2-antagonist PD123319	[18]
AT1-knockout mice	↑ BP in wild-type mice at ≈10× ↑ concentrations than Ang II no effect on BP in AT1_A_-knockout mice	[17]
	↑ BP and cortical vascular resistance and ↓ cortical blood flow in wild-type mice by Ang A and Ang II abolished in AT1_A_-knockout mice	[18]
AT2-knockout mice	↑ cortical vascular resistance and ↓ cortical blood flow inhibited by candesartan no effect of AT2-antagonist PD123319	[18]

**Table 2:** Biological effects of alamandine.

Alamandine		References
Human ACE2 (hACE2) cells	Forming of alamandine by ACE2	[22]
Isolated rat heart	Forming of alamandine after perfusion with Ang 1–7	
MrgD-transfected cells	Alamandine specifically binds to MrgD-cells abolished by AT2-agonist PD123319 No release induced by alamandine	
Aortic rings FVB/N mice, Mas-deficient mice AT2-knockout mice, Wistar rats	Endothelial-dependent vasorelaxation attenuated by pretreatment with NO-synthase antagonist L-NAME completely blocked by Ang 1–7 antagonist d-Pro^7^ -Ang-(1–7) not influenced by Mas antagonist A-779 preserved in AT2- and Mas-deficient mice inhibited by preincubation with â-alanine	
Aorta, iliac, carotid, and renal artery New Zealand White rabbits	No direct vasoactive effect, vasodilation mediated by acetylcholine ↑ acetylcholine-mediated vasodiation in aorta and iliac artery of healthy animals no effect on acetylcholin-mediated vasodilation in carotid artery ↓ acetylcholine-mediated vasodilation in the renal artery no vasoactive effect in vessels from animals fed with atherogenic diet ↓ Ang A-mediated vasoconstriction no effect on Ang II-mediated vasoconstriction	[20]
Fisher rats microinjection into rostral and caudal ventrolateral medulla	Rostral ventrolateral medulla–pressor effect caudal ventrolateral medulla–depressor effect blocked by Ang 1–7 antagonist D-Pro^7^-Ang-(1–7) not influenced by Mas antagonist A-779	[22]
Spontaneously hypertensive rats single dosis of alamandine	Long-term antihypertensive effect	
Isoproterenol-treated Wistar rats 50 µg/kg/day alamandine	↓ Collagen I, III, and fibronectin in the heart	
Sprague-Dawley rats intracerebral ventricular infusion	↑ Bradycardic component of the baroreflex	[24]
